# The association of inflammatory response with speckled global strain in recovered patients with myocarditis

**DOI:** 10.1097/MS9.0000000000004210

**Published:** 2025-10-30

**Authors:** Haitham Abu Khadija, Gilad Saar, Mohammad Alnees, Omar Ayyad, Amir Haim, Sara Shimoni, Jacob George, Sorel Goland

**Affiliations:** a Department of Cardiology, Kaplan Medical Center and Faculty of Medicine, Hebrew University of Jerusalem, Jerusalem, Israel; b Department of Nephrology, Samson Assuta Ashdod University Hospital, Ashdod, Israel; c Harvard Medical School, Postgraduate Medical Education, Global Clinical Scholer Research Training Program, Boston, US

**Keywords:** cardiac function, global longitudinal strain (GLS), inflammatory markers, myocarditis, neutrophil-to-lymphocyte ratio (NLR)

## Abstract

**Objectives:**

This study aimed to investigate the relationship between simple, routinely available inflammatory markers and residual impairment of left ventricular (LV) function assessed by 2D global longitudinal strain (GLS) in patients recovered from myocarditis.

**Materials and Methods:**

This prospective study included patients diagnosed with Myocarditis who were followed in our heart failure clinic. All participants underwent conventional echocardiography and 2D strain analysis at follow-up. Echocardiographic data was compared with age and sex-matched healthy controls. The study group’s bloodwork was analyzed for inflammatory biomarkers, including neutrophil-to-lymphocyte ratio (NLR), at defined time intervals starting from admission.

**Results:**

A total of 104 myocarditis patients [22 women, mean age 30.9 ± 8.5 years, baseline LV ejection fraction (LVEF) 56.8 ± 6.8%] were included. At a median follow-up of 78.4 months (IQR: 53.5–99.2), median GLS remained significantly less negative compared with healthy controls [–18.0% (IQR: −18.8 to −17.5) vs −21.4% (IQR: −22.7 to −19.4); *P* < 0.001]. At presentation, patients showed elevated inflammatory markers (WBC 10.7 ± 3.4 K/µL, NLR 5.53 ± 3.54, C-reactive protein 81.1 ± 67.7 mg/L), with WBC and NLR demonstrating an initial increase followed by significant decline at 72 hours and 6 months (*P* < 0.001). Regression analyses confirmed impaired GLS in myocarditis patients compared to controls (crude β = 2.88, 95% CI: 2.21–3.55; adjusted β = 2.54, 95% CI: 1.63–3.44; *P* < 0.001). Within the myocarditis cohort, admission NLR was independently associated with impaired GLS at follow-up (crude β = 0.190, 95% CI: 0.103–0.277; adjusted β = 0.170, 95% CI: 0.088–0.267; *P* < 0.001). Sex-stratified analysis showed significance in males (β = 0.179, 95% CI: 0.088–0.270; *P* < 0.001; R^2^ = 0.163) but not in females (β = 0.194, 95% CI: −0.107 to 0.496; *P* = 0.194; R^2^ = 0.083).

**Conclusion:**

Elevated inflammatory markers at presentation, particularly NLR, were associated with impaired GLS at follow-up, independent of baseline demographic and clinical covariates. The predictive value of NLR appeared more evident in males, though limited female representation precludes firm sex-specific conclusions. NLR may serve as a simple, cost-effective marker for identifying patients at risk of residual subclinical LV dysfunction after myocarditis, but larger, sex-balanced cohorts are required to confirm these findings.

## Introduction

Myocarditis is an inflammatory process involving the myocardium that has a broad etiology and includes infectious, toxic, and immune-mediated causes. Myocarditis has a variable clinical presentation ranging from asymptomatic or mild disease, which resolves spontaneously, to fulminant disease with consequent dilated cardiomyopathy that may lead to severe heart failure and poor outcomes^[1,2]^. In the vast majority of cases, the prognosis is generally good with recovery in most patients^[1]^. Given its diverse clinical features and overlap with other acute cardiac conditions, myocarditis diagnosis is challenging and is often overlooked^[2]^. According to the World Health Organization/International Society and Federation of Cardiology, as well as the 2013 European Society of Cardiology (ESC) myocarditis Task Force report, diagnosis of myocarditis is made by histological (Dallas criteria), immunological, and immune-histochemical criteria^[3,4]^. Most of the clinically stable patients with manifestations of myocarditis do not undergo endomyocardial biopsy. Therefore, a definitive diagnosis usually does not take place. The ESC criteria use the term “clinically suspected myocarditis” (CSM) to establish the diagnosis if ≥1 clinical presentation and ≥1 diagnostic criteria from different categories are present, in the absence of angiographically detectable coronary artery and known pre-existing cardiovascular disease or extra-cardiac causes that could explain the syndrome^[4]^. Cardiac magnetic resonance (CMR) currently serves as the gold standard imaging modality for the diagnosis and risk stratification of myocarditis. Although CMR is considered a more readily available imaging technique nowadays, it still poses a financial and operational burden on the healthcare system. Given the composite natural history and prognosis of myocarditis, there is a great interest in cost-effective techniques to is associated with the risk of future adverse outcomes better^[5]^. Current data indicate that left ventricular (LV) ejection fraction (LVEF) assessment via standard echocardiography lacks the sensitivity needed to reflect complete recovery of LV function accurately^[6,7]^. In contrast, TDI and 2D tracking imaging provide a more precise evaluation of LV function. The 2DS technique for quantification of myocardial strain provides data on longitudinal and circumferential myocardial function and rotation. These deformation indices, especially global longitudinal strain (GLS), were reported to be sensitive indicators of subtle changes in LV function^[6,7]^.

In addition to imaging techniques, the neutrophil-lymphocyte ratio a marker of systemic inflammation is gaining traction in cardiovascular research. The neutrophil-to-lymphocyte ratio (NLR) reflects the balance between neutrophil-mediated inflammation and the regulatory processes of lymphocytes, with increased NLR values associated with poorer outcomes in various cardiac conditions. Traditionally, absolute neutrophil counts have been linked to cardiovascular events, while absolute lymphocyte counts have shown negative associations^[8,9]^. While total white blood cell counts can is associated with cardiovascular risk, NLR demonstrates a superior predictive capability^[10]^. Some studies have assessed the NLR as a predictor of total mortality following acute coronary interventions and in heart failure contexts^[10–14]^. However, there is limited data on the clinical, laboratory, and echocardiographic course of myocarditis in adults, with some studies indicating a benign course, while others suggest a worsening prognosis^[15]^. Furthermore, no research has yet explored the relationship between NLR and cardiac imaging results alongside other known inflammatory markers in myocarditis patients. Given its representation of various systemic inflammation pathways, NLR could serve as a single, widely available inflammatory biomarker beneficial for routine clinical practice.

Our study aims to outline the clinical trajectory, recurrence, and LV function at follow-up in patients diagnosed with myocarditis. In addition, it will evaluate any potential relationship between the initial level of inflammatory markers and NLR measured during the acute phase of the disease and their effect on residual impairment of LV function expressed by change in GLS over follow-up. This study has been reported in line with the STROCSS guidelines^[16]^.

## Materials and methods

### Study design and population

This single-center prospective cohort study with a 1:2 matched case–control design was conducted at Kaplan Medical Center, Israel, between January 2010 and December 2021. A total of 182 participants were initially enrolled, including 125 patients diagnosed with clinically suspected myocarditis and 57 age-, sex-, and body mass index (BMI)-matched healthy controls. The diagnosis of myocarditis was established according to ESC criteria^[4]^. Due to limited availability of cardiac MRI during admission, 59 patients underwent MRI and fulfilled the Updated Lake Louise criteria^[4]^. All patients were subsequently followed in the outpatient heart failure clinic after hospital discharge.

**HIGHLIGHTS**

- Myocarditis patients showed significantly reduced global longitudinal strain (GLS) compared to healthy controls at follow-up.
- Higher neutrophil-to-lymphocyte ratio (NLR) at admission correlated with worse GLS, indicating residual cardiac dysfunction.
- These findings suggest that simple, routinely available blood tests may help predict long-term myocardial impairment in myocarditis survivors.

Inclusion criteria for the myocarditis group were: (1) age ≥ 18 years, (2) diagnosis of clinically suspected myocarditis according to ESC criteria patients underwent MRI, (3) availability of bloodwork and echocardiographic data at admission, and (4) at least 12 months of follow-up.

**Figure 1.**
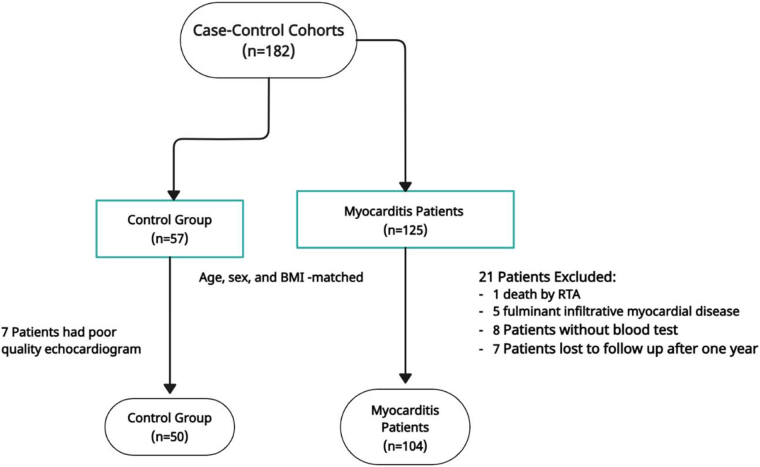
Study flowchart of patients from 2010 to 2021. RTA = road traffic accident.

For the control group, 7 individuals were excluded due to poor echocardiographic image quality, leaving 50 controls for final analysis. For the myocarditis group, 21 patients were excluded: 1 due to death from a road traffic accident, 5 due to fulminant infiltrative myocardial disease (2 cases of giant cell myocarditis and 3 cases of cardiac sarcoidosis), 8 patients without blood test results, and 7 patients lost to follow-up after 1 year. The final study cohort therefore consisted of 104 patients with myocarditis and 50 matched controls (Fig. 1).

Follow-up duration was calculated from the date of index hospitalization to the date of the last clinic visit or echocardiographic assessment. Among myocarditis patients, the median follow-up was 78.4 months (IQR: 53.5–99.2 months), with all patients having at least 12 months of follow-up. Healthy control participants underwent a single baseline echocardiographic examination without longitudinal follow-up.

In addition to the case–control comparison, we performed a focused longitudinal analysis restricted to the myocarditis cohort (*n* = 104), evaluating NLR at admission as a predictor of global longitudinal strain (GLS) measured 1 year after the index event. This complementary analysis was designed to specifically examine the prognostic role of inflammatory markers in predicting residual LV dysfunction among myocarditis patients.

### Literature search strategy

To support the study rationale and analytic choices, we conducted a targeted literature search of PubMed/MEDLINE, Embase, and Web of Science (coverage: January 2000 to December 2024) using prespecified query blocks combining myocarditis terms with deformation imaging and inflammatory indices (“myocarditis” OR “inflammatory cardiomyopathy”) AND (“global longitudinal strain” OR “GLS” OR “speckle-tracking” OR “2D-STE”) AND (“neutrophil-to-lymphocyte ratio” OR “NLR” OR “inflammatory marker*” OR “C-reactive protein” OR “CRP”), followed by title/abstract screening, full-text review of adult cohorts reporting GLS and/or inflammatory markers in myocarditis or related conditions, hand-searching of reference lists and guideline documents, and record management in Mendeley.

Situated at the confluence of standardized deformation-imaging protocols and pragmatic inflammatory phenotyping, our analytic framework deliberately integrates methodological precedents from two distinct lines of inquiry. First, a multicenter registry of immune-checkpoint-inhibitor myocarditis applied central, vendor-neutral core-lab quantification of LVGLS from the three apical views (TomTec), with the analyst blinded to clinical data; design elements included a retrospective case–control framework (101 cases; 92 ICI-treated controls without myocarditis), GLS measured at presentation, and a prespecified composite MACE endpoint over short-term follow-up thereby establishing standardized deformation-imaging procedures and outcome adjudication, albeit without long-horizon recovery imaging^[17]^. Second, a single-center retrospective cohort of 202 adults with clinically adjudicated myocarditis operationalized admission cell-count–derived indices (NLR, MLR) from routine laboratories and related them to severity proxies (e.g., length of hospital stays via correlation and ROC analyses) and LV systolic function, but did not incorporate deformation imaging or longitudinal echocardiographic follow-up^[18]^. Our study integrates these strands by prospectively specifying admission NLR as a novel predictor of 2D-STE–derived GLS at 1 year, in ESC-defined suspected myocarditis with obstructive CAD excluded and Lake Louise CMR applied when available thus linking an easily obtainable inflammatory biomarker at index presentation to quantitative myocardial deformation recovery at a fixed late time point, a question not addressed by prior designs^[17,18]^.

### Clinical and laboratory data, including inflammatory markers

Each patient was evaluated clinically by physical examination, Complete blood count including total white blood cell (WBC) counts and its differential, biochemical analyses such as renal parameters, lipid profile, and other markers of inflammation consisting of C-reactive protein (CRP), ESR, and LDH. Additional serological tests aimed at identifying the etiology of the disease were performed based on the initially presented features. All blood samples were examined and documented at hospital admission and follow-up visits. The NLR ratio was constructed as follows: NLR = neutrophil absolute count divided by lymphocyte absolute count.

### Control group assessment

Healthy control participants were recruited to provide reference echocardiographic values and were matched to myocarditis patients by age, sex, and BMI. By study design, control participants underwent a single transthoracic echocardiographic examination without laboratory phlebotomy or biomarker analysis. Thus, data on WBC counts, NLR, and CRP were not available for the control group.

### Updated Lake Louise (2018) CMR criteria

When performed, CMR adhered to the 2018 Updated Lake Louise criteria, diagnosing acute myocardial inflammation when ≥1 T2-based edema criterion and ≥1 T1-based non-ischemic injury criterion (elevated native T1 or extracellular volume, or a non-ischemic LGE pattern) were present. Pericardial effusion/enhancement and pleural effusions were treated as supportive findings. Acquisition parameters and interpretation followed contemporary consensus recommendations. The ULLC was applied when CMR data were available^[19,20]^.

### Echocardiography, 2D-STE, and additional imaging modalities

All patients underwent a detailed transthoracic echocardiographic examination at presentation and during follow-up 78.4 [53.5–99.18] months, performed by experienced technicians blinded to the study groups. Conventional echocardiographic measurements were recorded as per recommendations of the American Society of Echocardiography^[21]^. LV global longitudinal strain (LVGLS) was measured by 2D-STE to determine myocardial deformation. Measurements of LVGLS were performed offline by a single experienced cardiologist using a commercially available software program (GE Healthcare EchoPAC Version 202). If required, manual adjustments were made by the physician. The apical four–three and two-chamber images were used for LVGLS analysis, and the mean GLS was calculated by averaging the peak GLS values of the mentioned views. All echo measurements were compared with 40 age-, sex-, and BMI-matched healthy controls enrolled in this study. No consensus reference range for strain has yet been established in myocarditis. Thus, the cut-off range of LVGLS was accepted as −18 % as described in previous studies^[22]^. A value lower than −18 % of LVGLS was determined as impaired LVGLS. A cardiac CMR was performed based on availability. Coronary artery disease was excluded at admission using coronary angiography or cardiac computer tomography angiography in patients with coronary risk factors or questionable presentation, mandating the need for differential diagnosis with acute coronary syndromes.

### Statistical analysis

Continuous variables were expressed as mean ± standard deviation if normally distributed, and as medians with interquartile ranges (Q1–Q3) when non-normally distributed. Categorical variables were presented as frequencies and percentages. Normality of continuous variables was assessed using the Kolmogorov–Smirnov test. For group comparisons,(1) a two-sample *t*-test was used for normally distributed variables, and (2) the Mann–Whitney *U* test was applied for non-normally distributed variables. Comparisons of baseline characteristics and cardiac function measurements between healthy controls and myocarditis patients were conducted using the Mann–Whitney *U* test for continuous variables. All *P*-values were two-sided, with a significance threshold at *P* < 0.05.

During the design phase, myocarditis patients and healthy controls were matched on age, sex, and BMI to reduce baseline confounding. Potential confounders for multivariable analysis were selected based on prior evidence from the literature, clinical relevance to myocardial function^[6,23–25]^, and statistical associations observed in univariate analyses. Accordingly, we adjusted for demographic and lifestyle factors (age, sex, BMI, and smoking) as well as echocardiographic parameters known to influence GLS (LVESD, LVEF, E/A ratio, deceleration time, and posterior wall thickness).

Case–control comparisons were evaluated using multivariable regression models, while prognostic analyses among myocarditis patients were modeled with GLS at follow-up as the dependent variable and NLR at admission as the primary predictor. Effect modification by sex was further explored using sex-stratified models. To strengthen robustness and reduce the impact of non-normality, we performed non-parametric bootstrapping with 2000 replications, using a fixed random seed (12345) to generate bias-corrected standard errors and 95% confidence intervals. Models follow the guidelines of the Transparent Reporting of a Multivariable Prediction Model for Individual Prognosis or Diagnosis^[26]^. All analyses were conducted in STATA version 17 (StataCorp, College Station, TX, USA).

## Results

Table 1 presents the clinical characteristics of 104 patients at the index event (average age of 30.97 ± 8.47 years). The significant majority were male (78.9%) and 10.5% had a BMI greater than 30 kg/m^2^, and 14 participants (20.8%) had a history of smoking. High sensitivity troponin T (hsTnT) levels were remarkably elevated (9918.45 ± 10 141.68 μg/l).

**Table 1:** Baseline characteristics, comorbidities, and biomarker levels in Myocarditis patients

	Overall (*N* = 104)
Demographic data	
Age, y	30.97 ± 8.47
Male	82 (78.85)
BMI > 30 kg/m^2^	11(10.5)
History of smoking	14 (20.8)
Chronic disease	4 (6)
Presenting symptoms	
Chest pain	63(60.58)
Dyspnea	23(22.12)
Arrhythmia	11(10.58)
Laboratory	
Creatinine (mg/dl)	0.85 ± 0.12
C-reactive protein (CRP, mg/l)	42.54 ± 37.76
Erythrocyte sedimentation rate (ESR) (mm/h)	26.02 ± 13.96
LDH (Units/l)	401.76 ± 202.24
Bilirubin (mg/dl)	0.67 ± 0.24
High-sensitivity troponin (hsTnT, μg/l)	9918.449 ± 10 141.68
Hemoglobin (g/dl)	14.08 ± 1.14
White blood cell (WBC) count (K/µl)	10.22 ± 3.1
Platelet count (K/µl)	223.52 ± 58.00
Absolute Neutrophils (K/µl)	7.42 ± 2.92
Absolute Lymphocytes (K/µl)	1.74 ± 0.71
Neutrophil-to-lymphocyte ratio (NLR)	5.53 ± 3.54
TSH (mIU/ml)	1.31 ± 0.35

Data present in mean ± SD or (*n*, %).

Among the 104 patients diagnosed with myocarditis, 8 patients (7.6%) experienced recurrent episodes during the follow-up period, which had a median duration of 78.4 [53.5–99.2] months.

**Figure 2.**
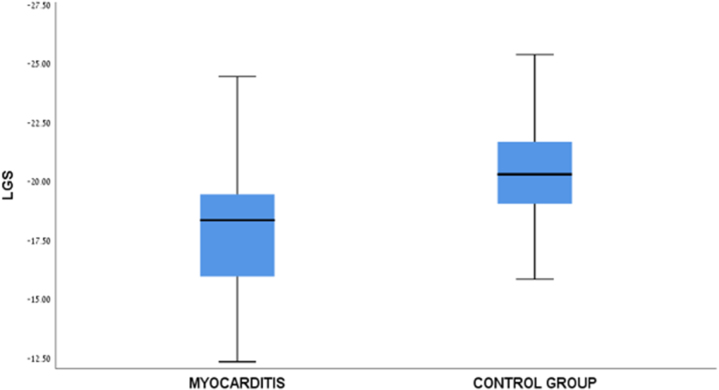
Comparison of mean average longitudinal global strain in myocarditis-recovered patients versus healthy controls.

Table 2 presents a comparison of baseline characteristics between patients with myocarditis (*n* = 104) and healthy age-, sex-, and BMI-matched controls (*n* = 50). At follow-up, mean LVEF in patients with myocarditis was slightly lower than that in healthy controls (56.75 ± 6.80 % vs 58.72 ± 1.77 %, *P* = 0.05). Moreover, the median GLS was significantly lower in the myocarditis group compared to the controls [–18.0 (–18.75 to −17.5) vs −21.4 (–22.7 to −19.4) %, *P* < 0.001; Figure 2].

**Table 2:** Comparison of characteristic and echocardiographic variables between the study group and control group

	Myocarditis patients (*n* = 104)	Healthy control (*n* = 50)	*P*-value
Age (y)	30.97 ± 8.47	30.52 ± 4.17	0.722^b^
Body mass index (kg/m^2^)	25.61 ± 2.99	25.15 ± 2.07	0.336^b^
LVEDD (mm)	46.009 ± 4.97	45.34 ± 2.98	0.381^b^
LVESD (mm)	30.80 ± 4.50	30.08 ± 3.02	0.302^b^
Left atrial diameter (mm)	34.115 ± 3.64	34.38 ± 2.56	0.645^b^
IVS thickness (mm)	9.25 ± 1.01	9.14 ± 0.80	0.466^b^
Posterior left ventricular wall thickness (mm)	8.5 ± 0.99	8.28 ± 2.45	0.169^b^
LVEF (%)	56.75 ± 6.80	58.72 ± 1.77	0.05^b^
Deceleration time (ms)	184.75 ± 45.92	164.38 ± 60.17	0.318^b^
Left ventricle E/e’	4(4–4)	5 (4.92–5.13)	<0.001^c^
E/A	2(1.69–2)	1(1–1.44)	<0.001^c^
GLS (%)^a^	−18(−18.75 to −17.5)	−21.4 (−22.7 to −19.4)	<0.001^c^

BMI, body mass index; E/e’, E-wave to e’ wave ratio; LVEDD, left ventricular end-diastolic diameter; LVESD, left ventricular end-systolic diameter; LVEF, left ventricular ejection fraction; MR, mitral regurgitation; GLS, global longitudinal strain; NLR, neutrophil-lymphocyte ratio; TR, tricuspid regurgitation.

Data presented in mean ± SD.

^a^ Data presented median (Q1–Q3).

^b^ Two-sample *t*-test.

^c^ Mann–Whitney *U* test.

Additionally, both echocardiographic diastolic measures were significantly different between the two groups, but were within normal ranges. The E/e’ was notably higher in myocarditis patients than controls [5 (4.92–5.13) vs 4 (4–4), *P* < 0.001]. Similarly, the E/A ratio was significantly decreased in myocarditis patients compared to controls [1 (1–1.44) vs 2 (1.69–2), *P* < 0.001].

**Figure 3.**
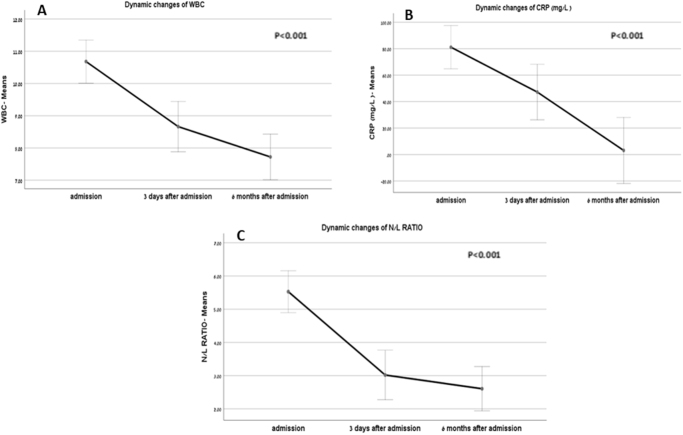
Dynamic changes of inflammatory components for the entire cohort, (A) dynamic changes in total WBC with time; (B) dynamic changes of CRP with time; (C) dynamic changes of N/L ratio with time. WBC, white blood cells (K/µl); N/L ratio, neutrophils to lymphocyte ratio; CRP, C-reactive protein.

The dynamic changes in inflammatory markers, including WBC counts, NLR, and CRP levels, were assessed at three distinct time points: presentation, 72 hours post-presentation, and at the 6-month follow-up. At presentation, the mean WBC count was 10.68 ± 3.42 K/µl, which significantly decreased to 8.66 ± 2.36 K/µl by 72 hours. Further, he declined to 7.72 ± 2.26 K/µl at the 6-month check-up, with a statistically significant *P*-value of less than 0.001 for the change between presentation and 6 months. The NLR was elevated at 5.53 ± 3.54 at presentation, significantly reducing to 2.66 ± 1.78 at 72 hours and continuing to 2.23 ± 1.16 by 6 months, again with a *P*-value of less than 0.001. Furthermore, CRP levels were markedly high at 81.1 ± 67.7 mg/l upon presentation, decreasing to 47.19 ± 37.22 mg/l at 72 hours, and reaching 3.08 ± 1.12 mg/l by the 6-month follow-up (Fig. 3).

As shown in Table 3, patients with clinically suspected myocarditis had significantly less negative GLS values compared with healthy controls. In crude analysis, the mean difference in GLS between groups was 2.88% (95% CI: 2.21–3.55; *P* < 0.001). This association remained robust after adjustment for smoking, LVESD, LVEF, E/A ratio, deceleration time, and posterior wall thickness, with an adjusted mean difference of 2.54% (95% CI: 1.63–3.44; *P* < 0.001). These findings indicate that myocarditis patients exhibit impaired myocardial deformation relative to matched controls, independent of conventional echocardiographic parameters and clinical covariates.

**Table 3:** Crude and adjusted regression of GLS between Myocarditis patients and healthy controls

Group	Model	β (95% CI)	*P*-Value
Healthy controls (*n* = 50)	Reference	–	–
CSM patients (*n* = 104)	Crude	2.88 (2.21–3.55)	<0.001
	Adjusted^a^	2.54 (1.63–3.44)	<0.001

CSM, clinically suspected myocarditis; GLS, global longitudinal strain.

^a^ Adjusted for smoking, E/A ratio, deceleration time, and posterior left ventricular wall thickness, left ventricular ejection fraction, left ventricular end-systolic diameter.

**Figure 4.**
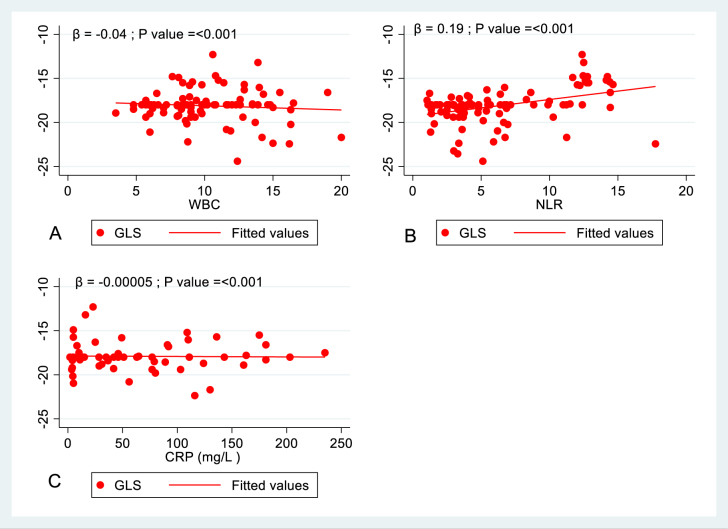
Univariate linear regression analysis of inflammatory markers with global longitudinal strain, (A) linear regression of WBC with GLS, (B) linear regression of NLR with GLS, (C) Linear regression of CRP with GLS. CRP, C-reactive protein (mg/l); GLS, global longitudinal strain; WBC, white blood cells (K/µl); NLR, neutrophils to lymphocytes ratio.

As shown in Table 4, higher NLR at admission was significantly associated with impaired GLS at follow-up in crude analysis (β = 0.190, 95% CI: 0.103–0.277, *P* < 0.001). This association remained robust after adjustment for age, sex, BMI, and smoking status (β = 0.170, 95% CI: 0.088–0.267, *P* < 0.001). These findings indicate that the prognostic value of NLR in predicting residual subclinical LV dysfunction is independent of baseline demographic and clinical covariates. The univariate analysis was performed for each inflammatory marker separately to assess a possible association with GLS, this association with GLS was obtained in WBC count and CRP values [(*P* < 0.001, β = −0.04) and (*P* > 0.001, β = −0.0005) respectively]. Figure 4 demonstrates the linear regression analysis.

**Table 4:** Crude and adjusted linear regression of NLR at admission in relation to GLS among Myocarditis patients (*N* = 104)

Model	Variable	β (95% CI)	*P*-value
Crude	NLR at admission	0.190 (0.103, 0.277)	<0.001
Adjusted^a^	NLR at admission	0.170 (0.088, 0.267)	<0.001

**β**, regression coefficient; CI, confidence interval; GLS, global longitudinal strain; NLR, neutrophil-to-lymphocyte ratio.

^a^ Adjusted for age at admission, sex, body mass index, and smoking status.

As shown in Table 5, NLR was significantly associated with impaired GLS in males (*n* = 81; β = 0.179, 95% CI: 0.088–0.270, *P* < 0.001, R^2^ = 0.163). In contrast, the association between NLR and GLS did not reach statistical significance in females (*n* = 22; β = 0.194, 95% CI: −0.107–0.496, *P* = 0.194, R^2^ = 0.083). These findings indicate that the predictive role of NLR for subclinical LV dysfunction after myocarditis appears more evident in males, while the limited female sample size precludes firm conclusions regarding sex-specific differences.

**Table 5:** Sex-stratified linear regression of GLS on NLR

Sex	Variable	β (95% CI)	*P*-Value	R^2^
Males (*n* = 81)	NLR	0.179 (0.088, 0.270)	<0.001	0.163
	Constant	–19.127 (–19.838, −18.417)	<0.001	
Females (*n* = 22)	NLR	0.194 (0.107, 0.496)	0.194	0.083
	Constant	–19.707 (–21.415, −17.999)	<0.001	

**β**, regression coefficient; CI, confidence interval; GLS, global longitudinal strain; NLR, neutrophil-to-lymphocyte ratio.

Linear regression models were fitted separately for males and females.

## Discussion

For the first time, our study showed that the degree of elevated inflammatory markers at the presentation of acute myocarditis, namely NLR, is strongly associated with altered 2D strain at follow-up, suggesting that a marked inflammatory response impacts residual impairment of LV function after recovery.

Notably, we observed a significant increase in WBCs, predominantly neutrophils, alongside a decrease in lymphocyte counts at presentation, leading to an elevation in the NLR. It is well-established that neutrophil counts are impacted by stress-induced release of hormones such as cortisol, epinephrine, and norepinephrine, which can lead to neutrophilia^[27]^. Conversely, the same stress hormones can cause lymphocytes to redistribute to lymphatic tissue, precipitating lymphopenia. Thus, NLR serves as a rapid and sensitive marker for physiological stress, reflecting systemic inflammation, including in cases of myocarditis^[28,29]^.

Neutrophils mediate inflammatory responses to acute injury through various biochemical mechanisms, resulting in further tissue damage. This includes the release of arachidonic acid metabolites, platelet-aggregating factors, cytotoxic oxygen-derived free radicals, and several hydrolytic enzymes. Such tissue damage can impair cardiac function over time. After the initial acute inflammatory response resolves, we noted a gradual decrease in NLR and other inflammatory markers (e.g., CRP) during the 6-month follow-up, yet laboratory exams cannot currently quantify myocardial damage without a reduction of ejection fraction as observed in echocardiography or CMR^[30–34]^.

Several investigations have documented subclinical cardiac impairment in myocarditis patients, manifested as reduced GLS values despite maintaining normal LVEF. Our study employed 2D echocardiographic GLS analysis to explore residual contractile changes in the left ventricle. Impaired deformation indexes were reported in patients recovered from peripartum cardiomyopathy and patients after the acute event of Takotsubo cardiomyopathy (6,7). In line with these reports, we demonstrated significantly lower GLS values than healthy controls in our patient population after acute myocarditis. This reduction correlates with findings from previous studies indicating that impaired GLS is a reliable indicator of subclinical myocardial dysfunction^[33,35,36]^.

A proposed mechanism for these findings may relate to the uneven involvement of different contractile fibers within the heart. Specifically, subendocardial fibers, which govern longitudinal LV functions, are more susceptible to damage, while radial functions, which rely on mid-myocardial and epicardial fibers, may remain relatively intact. This discrepancy allows for a preserved LVEF, masking the underlying dysfunction identified by GLS measurements. Additionally, we observed that the LVESD was significantly higher in myocarditis-recovered patients compared to controls, suggesting potential myocardial damage and tissue remodeling induced by prior inflammation. This aligns with findings suggesting that echocardiographic increases in LVESD can is associated with changes in LVEF in heart failure patients^[33,37,38]^.

These echocardiographic alterations could foreshadow the severity and extent of future myocardial dysfunction, highlighting the need for vigilant monitoring in this population^[35,39]^.

Intriguingly, univariate analysis indicated that NLR at presentation was more strongly associated with impaired GLS than established markers such as CRP and WBCs. This underscores the clinical relevance of NLR as a readily obtainable test in routine practice. Our findings suggest that NLR could serve as a valuable biomarker for risk stratification in myocarditis patients, aligning with previous research linking inflammatory markers to disease severity and prognosis in various cardiovascular conditions^[34,40–43]^.

Furthermore, the GLS values obtained in this study confirmed the presence of subclinical cardiac dysfunction, revealing a statistically significant difference between the myocarditis group and healthy controls. This supports the idea that GLS is a superior measure of LV function compared to LVEF, particularly in low-risk populations.

### Addressing the imbalance between cases and controls

One of the limitations of our study was the unequal ratio between myocarditis patients and healthy controls. To minimize the impact of this imbalance, we applied several complementary methodological approaches. First, we matched patients and controls on age, sex, and BMI, which reduced baseline differences in key demographic factors. Second, because matching alone may not fully eliminate residual confounding, we employed multivariable regression to further adjust for clinical and echocardiographic covariates such as smoking status, LVESD, LVEF, E/A ratio, deceleration time, and posterior wall thickness. Finally, we implemented bootstrapping with repeated resampling to obtain more stable confidence intervals and ensure the robustness of our estimates. By combining these strategies, we believe that the observed associations are unlikely to be explained by the unequal case-to-control ratio, thereby strengthening the internal validity of our findings.

### Sex stratification and control of confounders

We also explored potential sex-related differences by conducting sex-stratified regression analyses. While the association between NLR and impaired GLS was statistically significant in males, it did not reach significance in females, most likely due to the small number of female participants in our cohort. This limitation reduces the power to draw firm sex-specific conclusions. Nevertheless, when accounting for major baseline covariates including age, sex, BMI, and smoking status in multivariable regression, the association between NLR and GLS remained robust and statistically significant. Taken together, these results highlight that although sex imbalance represents an important limitation of our study, the independent predictive role of NLR was preserved after adjusting for key confounders, thereby strengthening the validity of our findings. An additional methodological strength of our study lies in the careful application of inclusion and exclusion criteria. For example, we excluded patients with infiltrative myocardial diseases such as giant cell myocarditis and cardiac sarcoidosis. These conditions follow a different clinical trajectory and pathophysiological process compared to viral or immune-mediated myocarditis, and their inclusion could have introduced heterogeneity and bias into the analysis. By excluding such cases, we aimed to ensure a more homogeneous study population and reduce confounding effects that might obscure the specific relationship between NLR and residual LV dysfunction assessed by GLS.

### Clinical implications of inflammatory markers

The present study highlights the potential prognostic role of inflammatory markers, particularly the NLR, in patients with myocarditis. Elevated NLR at admission was independently associated with impaired GLS at long-term follow-up, even after adjustment for conventional risk factors and echocardiographic variables. This suggests that NLR could serve as a simple, cost-effective, and widely available biomarker for risk stratification, helping to identify patients at higher risk of persistent subclinical LV dysfunction after recovery from acute myocarditis. While our findings support the integration of inflammatory markers into prognostic assessment, validation in larger and more diverse cohorts is required before routine clinical application can be recommended.

### Limitations

While our study provides important insights, several limitations should be acknowledged. First, although the sample size is adequate for preliminary observations, it remains relatively small, which may limit the generalizability of the findings. Nonetheless, all patients were followed for at least 1 year, ensuring comprehensive data collection and robust longitudinal assessment. Second, not all patients underwent CMR at our institution, primarily due to limited access to imaging resources, which may have restricted the depth of structural and tissue characterization. Third, the variable follow-up duration among myocarditis patients, which may have introduced heterogeneity in the timing of outcome assessment. Nevertheless, all patients had at least 1 year of follow-up, and the median follow-up period exceeded 6 years, providing substantial longitudinal data to support the validity of our findings. In contrast, healthy controls underwent a single baseline echocardiographic examination, which precluded longitudinal comparison between groups. Finally, healthy control participants did not undergo blood sampling, and thus inflammatory markers such as WBC count, NLR, and CRP could not be directly compared with those of myocarditis patients. This was an intentional design choice, as controls were enrolled to provide echocardiographic reference values only. While this does not compromise our primary objective, focusing on the prognostic role of inflammatory markers within the myocarditis cohort. it precludes side-by-side biomarker comparisons across groups. Future studies integrating both echocardiographic and laboratory data in control participants would provide additional and valuable comparative insights.

## Conclusion

The degree of elevated inflammatory markers at presentation, particularly NLR, was strongly associated with impaired GLS at long-term follow-up. This relationship remained significant after adjustment for age, sex, BMI, and smoking status, suggesting that NLR may serve as an independent predictor of residual subclinical LV dysfunction in patients recovering from myocarditis. While our sex-stratified analysis indicated that this association was more evident among males, the small number of female participants limited definitive conclusions regarding sex-specific differences. Therefore, NLR may aid in the early identification of high-risk patients, but larger, more sex-balanced studies are warranted to validate these findings and strengthen their generalizability.

## Data Availability

The data that support the findings of this study are not publicly available due to privacy and ethical restrictions according to the Declaration of Helsinki and local hospital regulations. However, data are available from the corresponding author upon reasonable request.

## Research registration unique identifying number (UIN)

This study was not registered with a clinical trial registry as it was an observational cohort without an intervention, but the authors recognize the importance of registration for prospective studies.
